# Metataxonomic Analysis and Fatty Acid Profiling of Feces from Children Undergoing Hematopoietic Stem Cell Transplantation

**DOI:** 10.3390/ijms27052331

**Published:** 2026-03-02

**Authors:** Claudio Alba, Laura Palomino, Beatriz Vergara, Marta Velasco Rodríguez-Belvis, Alberto Aragón, Marianna A. Di Campli Zaghlul, Rubén Jurado, Carmen Martín-Fernández, Julio A. Vázquez-Gómez, Marta González-Vicent, Blanca Molina-Angulo, Paula Sánchez-Llorente, Paloma García-Hernández, Juan M. Rodríguez, Rosa A. Muñoz-Codoceo, Carmen Herranz

**Affiliations:** 1 Department of Nutrition and Food Science, Complutense University of Madrid, 28040 Madrid, Spain; jmrodrig@ucm.es; 2 Instituto Pluridisciplinar, Complutense University of Madrid, 28040 Madrid, Spain; 3 Department of Gastroenterology and Nutrition, Hospital Infantil Universitario Niño Jesús, 28009 Madrid, Spain; laura.palomino.perez@gmail.com (L.P.);; 4 Department of Oncology, Hospital Infantil Universitario Niño Jesús, 28009 Madrid, Spain; 5 Department of Galenic Pharmacy and Food Technology, Complutense University of Madrid, 28040 Madrid, Spain

**Keywords:** hematopoietic stem cell transplantation, microbiome, microbiota, feces, *Blautia*, short-chain fatty acids, butyrate, acetate

## Abstract

Allogeneic hematopoietic stem cell transplantation (HSCT) is a medical procedure to treat hematologic malignancies and restore bone marrow function. However, this approach may lead to graft-versus-host disease (GvHD), a major cause of mortality and morbidity after allogeneic HSCT. Some studies have suggested the involvement of gut microbiota in the development and prognosis of GvHD. In this context, the main objective of this study was to compare the fecal microbiome composition and short-chain profile of pediatric patients who underwent successful HSCT, developed GvHD or died. The bacterial composition was analyzed using 16S rRNA gene sequencing, while short-chain fatty acids (SCFAs) were quantified by gas chromatography. Fecal samples at engraftment were mainly characterized by a loss of bacterial diversity, a depletion of sequences belonging to the genus *Blautia* and significantly lower concentrations of fecal butyrate and acetate compared with those obtained before HSCT and 100 days after HSCT. Our findings confirm that children experiencing GvHD after HSCT have distinct gut microbiota and SCFA profiles, which might contribute to developing new microbiota-targeted strategies for GvHD prevention during HSCT procedures.

## 1. Introduction

Hematopoietic stem cell transplantation (HSCT) is a medical procedure to treat hematologic malignancies, such as leukemia or lymphoma, and to restore bone marrow function in patients with conditions associated with dysfunctional hematopoiesis, such as aplastic anemia [1]. In autologous HSCT, hematopoietic stem cells are collected from the patient itself (i.e., the patient acts as their own donor) and reinfused after cytoreductive conditioning. This strategy has the advantage of avoiding host rejection, but might not be feasible in conditions such as leukemia, where the reinfused cells may be contaminated by malignant cells. Allogeneic HSCT, involving the collection of hematopoietic stem cells from a genetically matched, healthy donor, is usually the alternative in such cases. However, this approach has the risk of leading to graft-versus-host disease (GvHD), a condition in which the grafted immune cells recognize the host’s normal tissues as foreign [2,3]. This disease can cause a variety of clinical manifestations, including diarrhea, abdominal pain, nausea, vomiting, wasting, liver dysfunction, oral ulcers, dermatitis, skin sclerosis, and others [1,4]. Despite the preventative measures developed, GvHD remains a major cause of mortality and morbidity after allogeneic HSCT [5,6,7].

The involvement of gut microbiota in the development and prognosis of GvHD is becoming increasingly evident in recent studies [7,8]. Host gut microbiome may be disrupted during the immunosuppressive treatment used in the conditioning procedure, aimed to ensure proper transplant progression and prevent GvHD [5]. Failure in recovering the microbiota diversity existing before conditioning has been linked to an increased post-HSCT mortality, which supports the important role of the gut microbiome in HSCT outcome [6]. However, the bacterial taxa involved in the microbial shifts observed in GvHD cases are not clearly established and differ among studies. The levels of some bacterial components (e.g., lipopolysaccharides) or metabolites, such as short-chain fatty acids (SCFAs), may also play direct or indirect roles either in the development or prevention of GvHD [7].

In this context, the main objective of this study was to compare the fecal microbiome composition and SCFAs profile of pediatric patients who underwent successful HSCT, developed GvHD or died. Such an approach may help in finding biomarkers for detecting patients with a higher predisposition to developing GvHD, which in turn may contribute to designing alternative clinical procedures, resulting in an improved prognosis.

## 2. Results

### 2.1. Patients’ Demographic Characteristics

A total of 63 patients were recruited in the study. Of these, four patients dropped out before sampling started. The main demographic characteristics of the remaining 59 participants are summarized in Table 1. Overall, the patients consisted of 58% males and 42% females, with a median age of 11.91 years (range: 3.50–21.22 years). A total of 83.1% of the patients (49/59) received cyclosporine as part of their immunosuppressive regimen. The group of patients that did not develop GvHD (*n* = 45; 76%) comprised 58% males and 42% females, with a median age of 12.13 years (range: 3.65–21.22 years), whereas the group of patients that developed GvHD (*n* = 14; 24%) comprised 57% males and 43% females, with a median age of 11.89 years (range: 3.50–16.36 years). Finally, the group of patients who died (*n* = 14; 24%) consisted 36% males and 64% females, with a median age of 11.51 years (range 6.84–17.46).

### 2.2. Metataxonomic Analysis

A total of 155 fecal samples were collected in this study (preH = 58; postH1 = 52; postH2 = 38; GvHD = 7), which yielded 141 sequences after metataxonomic analysis. However, two sequences were excluded from further analysis due to their low quality. Overall, the remaining 139 samples (preH = 53; postH1 = 43; postH2 = 37; GvHD = 6) yielded a total of 37,242,101 high-quality paired-end reads. The number of reads per sample ranged from 85,608 to 692,008, with a median value of 212,223 (IQR: 180,838–285,590.5).

The fecal bacterial diversity (Amplicon Sequence Variants, ASVs) assessed using the Shannon diversity index revealed significant differences between preH samples (median [IQR] = 3.15 [2.58–3.73]) and postH1 samples (2.38 [1.7–3.37]) (*p* = 0.03) (Table 1). Overall, this index increased in postH2 samples (3.37 [3.00–3.71]), which were significantly different from both postH1 (*p* < 0.001) and GvHD (2.00 [1.31–2.55], *p* < 0.05).

The assessment of α-diversity using the Simpson index provided a very similar, although less significant, pattern. The values in preH (0.92 [0.86–0.95]) and postH1 (0.85 [0.7–0.94]) did not differ significantly. In contrast, postH2 (0.93 [0.89–0.95]) values were significantly higher than postH1 (*p* < 0.001), while the *p*-value for postH2 (0.93 [0.89–0.95]) and GvHD (0.82 [0.63–0.89]) comparison was 0.050 (Table 2).

**Figure 1:**
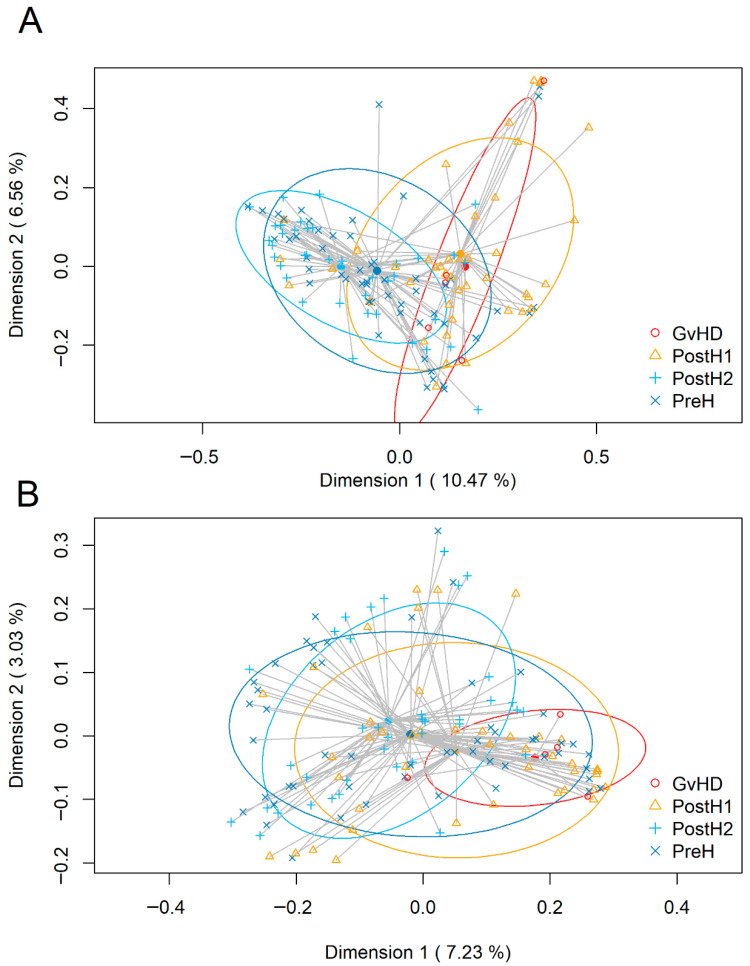
β-diversity, represented as a principal coordinate analysis (PCoA), of the fecal samples collected at the different sampling points: preH (day 0; dark blue crosses); postH1 (day 7; orange triangles); postH2 (day 28; light blue pluses); and GvHD (red circles). (**A**) Bray–Curtis dissimilarity; (**B**) Jaccard distance. Axes show the percentage of total variance captured by each dimension. Colored ellipses include the 50% confidence region around each group centroid. Grey lines link each sample with its corresponding centroid.

Subsequently, beta diversity was assessed using principal coordinate analysis (PCoA) applied to Bray–Curtis and Binary Jaccard distance matrices (Figure 1). The Bray–Curtis PERMANOVA analysis indicated significant differences in community structure among sampling points (Figure 1A). Pairwise comparisons with Bonferroni correction showed that postH2 bacterial communities differed significantly from the postH1 (*p* = 0.006) and GvHD (*p* = 0.010) ones; in addition, the preH communities were also significantly different when compared to the postH1 ones (*p* = 0.006).

Similar patterns were observed using the Jaccard index (Figure 1B), a parameter that focuses on bacteria detection irrespectively of relative abundance. The samples collected from the postH2 time point showed statistically significant (*p* < 0.01) differences when compared to those obtained from postH1 and GvHD sampling points.

A total of 28 phyla and 460 genera were identified among the reads obtained from the samples analyzed in this study. Bacillota, Pseudomonadota, Bacteroidota and Actinomycetota were the dominant phyla in all sampling points (Table 3). Phylum-level comparisons indicated that Bacillota, Pseudomonadota, Bacteroidota, and the “Minor_phyla” group did not show statistically significant differences in their relative abundance among the sampling points (Table S1). In contrast, statistically significant differences were observed in relation to the phylum Actinomycetota, which showed a lower relative abundance in the GvHD sampling point when compared with the other three groups (*p* < 0.012) (Table S1).

Differences in the relative abundance of some bacterial genera were also detected when comparing the four sampling points. Most of the shifts were related to genera belonging to the phylum Bacillota and, among them, those affecting the genus *Blautia* were particularly relevant. This genus was one of the few (together with the genera *Streptococcus* and *Enterococcus*) that was detected in 100% of the samples obtained in all the sampling points (Table 3). In addition, its median [IQR] relative abundance was the highest for a single genus in the preH (8.38 [2.45–17.89]) and postH2 (14.62 [6.36–26.13]) sampling points. This abundance decreased significantly in postH1 samples (0.23 [0.03–4.58)]) (*p* = 0.001), and then significantly increased in the postH2 samples (*p* < 0.001) (Table 3 and Table S1). At the GvHD sampling point, the relative abundance of *Blautia* spp. (0.02 [0.01–0.04]) was significantly lower than that of the preH and postH2 sampling points (*p* ≤ 0.020) (Table S1). On the other hand, the most abundant genus in the GvHD sampling point was *Streptococcus* (13.52 [3.41–25.09]), which was also true for postH1 samples (4.53 [0.43–19.65]). In fact, this genus was the only one, together with *Enterococcus*, which means relative abundance increased from preH to postH1 samples.

Although the frequency of detection, and particularly the relative abundance, of other members of the phylum Bacillota were lower than those of *Blautia* spp., significant differences were also observed in their relative abundances among the sampling points (Table 3 and Table S1). In this respect, the relative abundance of *Thomasclavelia* was lower in postH1 than in preH (*p* = 0.031) and postH2 (*p* < 0.001) samples, as well as in postH2 than in GvHD (*p* = 0.022). A similar pattern was observed for the *Ruminococcus gnavus_group* between postH1 and postH2 samples (*p* = 0.002), while the abundance of *Anaerostipes* was lower in postH1 samples (*p* = 0.001) and GvHD samples (*p* = 0.034) than in preH. Lower relative abundances were also found for the genus *Romboutsia* in postH1 than in preH (*p* = 0.007) and postH2 samples (*p* < 0.001), and in GvHD than postH2 (*p* = 0.007).

The relative abundance of the phylum Pseudomonadota was much lower than that of the phylum Bacillota at all sampling points. Within the phylum Pseudomonadota, *Escherichia*/*Shigella* displayed a higher relative abundance in postH2 vs. postH1 samples (*p* = 0.047); conversely, the genus *Ralstonia* had a higher relative abundance in postH1 than in preH [*p* = 0.006] and postH2 samples [*p* < 0.001]).

When the *α*- and *β*-diversity at each of the sampling points were compared between patients that developed GvHD or not, no differences were observed between these groups. However, differences were detected in the relative abundance of some of the genera present in the preH sampling point between both types of patients. Namely, the relative abundance of the genera *Bacteroides* and *Parabacteroides* in the preH samples was significantly lower (*p* < 0.05) in the patients that later developed GvHD (<0.01 [<0.01–0.56] and <0.01 [<0.01–<0.01], respectively), than in those that did not (1.4 [<0.01–5.09] and <0.01 [<0.01–0.48], respectively). However, the opposite was true for *Klebsiella* spp., which showed a higher relative abundance (*p* = 0.03) in the preH samples of the GvHD patients (0.01 [0.01–0.07]) than that of the non-GvHD group (0.01 [<0.01–0.01]).

On the contrary, differences were observed in the bacterial *α*-diversity of both the preH and postH1 samples of the patients who survived or died after HSCT. Specifically, the *α*-diversity was higher (*p* < 0.05) in the preH and postH1 samples of the survivors (3.42 [2.85–3.97]) and 2.89 [1.82–3.53], respectively) compared with that of the *exitus* group (2.88 [2.36–3.13]) and 1.86 [1.61–2.13], respectively). No differences between the two groups of patients were observed either in their *β*-diversity, or in the relative abundance of each bacterial genus.

### 2.3. SCFA Analysis

A total of 153 fecal samples collected in this study (preH = 55; postH1 = 53; postH2 = 41; GvHD = 4) were subjected to SCFA analysis. The median [IQR] values of acetic, butyric and propionic acids are shown in Table 4, while the complete data for these and other SCFAs (caproic, isobutyric, isovaleric and valeric acids) detected in the four sampling points analyzed in this study are presented in Table S2; in addition, a heat map representing the seven SCFAs according to the sampling point and GvHD/exitus status is shown in Figure S1.

In the preH samples, the median values for acetate, butyrate and propionate were 2005.8, 803.9 and 477.1 μg/g, respectively (Table 4). A sharp decrease in the levels of these three SCFAs was observed in the postH1 samples (524.7, 21.6 and 64.4 μg/g, respectively). The differences between these two time points were statistically significant for the three SCFAs (*p* < 0.001) (Table 4). In the postH2 samples, the median values of acetate, butyrate, caproate, isobutyrate, isovalerate and valerate had returned to values similar (*p* > 0.05) to those found in the baseline (preH) samples; however, propionate showed a mean value (821.4 μg/g) significantly higher (*p* = 0.02) than preH samples (Table 4 and Table S2). However, in the case of the samples from the GvHD sampling point, the levels of these three SCFAs were significantly lower (585.4; 3.0 and 84.0 μg/g, respectively) than those found in the postH2 samples (*p* < 0.05) and also the preH acetate and butyrate levels (Table 4).

When all the samples obtained from patients that developed GvHD were compared to those who did not suffer such a condition, the median values for acetate (585.4 and 1762.2 μg/g, respectively), butyrate (108.5 and 430.9 μg/g, respectively) and propionate (455.4 and 179.8 μg/g, respectively) were significantly higher in the samples provided by GvHD-negative patients (*p* < 0.05) (Table S2; Figure S1). Moreover, by combining the “sampling point” and the “GvHD” categories, it was found that patients who later developed GvHD displayed lower concentrations of acetate, butyrate and propionate in the preH samples, but the differences were not statistically significant (Figure S1). Samples from postH1 patients showed the same trend, although in this case the acetate concentration was significantly higher (*p* = 0.034) in the non-GvHD (602.0 [215.7–1735.2] μg/g) than in the GvHD group (131.2 [20.9–549.8] μg/g) (Table S2; Figure S1).

Similarly, when the all the samples obtained from patients who died during the study were compared to those who survived the HSCT process, the median values for acetate (811.7 and 1762 μg/g, respectively), butyrate (73.5 and 451.20 μg/g, respectively) and propionate (166.0 and 477.1 μg/g, respectively) were significantly higher in the samples provided by surviving patients (*p* < 0.01) (Table S2; Figure S1). Finally, the combination of “sampling point” and “exitus” categories did not show significant differences in the concentrations of these SCFAs.

## 3. Discussion

In this work, a prospective longitudinal study was carried out in a pediatric population suffering from different hematological disorders and assigned for allogeneic HSCT. Fecal samples from these patients were analyzed to determine their microbial and SCFA composition over time.

Allogeneic HSCT is a life-saving procedure that uses preparative regimens consisting of chemotherapy, radiotherapy, immunotherapy, or combinations thereof to establish a competitive advantage for donor hematopoietic cells. Such treatments have the potential to alter the gut microbiota of the host [9,10,11,12]. In fact, a significant temporary disruption of the gut microbiota, characterized by a shift in bacterial α- and β-diversity indices when compared to the time prior to the HSCT process, has been observed in this work and in previous studies [1,13,14,15]. In addition, the inability to drive the diversity and composition of the gut microbiota to basal levels in the following weeks after the HSCT procedure has been linked to the development of acute or chronic GvHD, a life-threatening complication of allogenic HSCT [16,17,18,19,20]. The relevant role of microbial diversity in preventing or decreasing the risk of GvHD is highlighted by the fact that patients with low, medium or high bacterial diversity indices in their gut microbiomes at the time of engraftment had survival rates of 36%, 60% and 67%, respectively, 3-year post HSCT [21].

Finding microbiome-related biomarkers of a specific condition or disease by using metataxonomic approaches is an elusive task because most studies include a relatively small cohort of patients and/or the results are often different (and sometimes even contradictory) between studies. However, the field of HSCT and GvHD seems to be an exception, since most studies carried out so far with different cohorts and in different geographic scenarios agree that a depletion in the relative abundance of *Blautia* spp. occurs in GvHD patients, as well as that harboring increased abundances of this genus is associated with reduced GvHD severity, lethality or improved overall survival in HSCT cohorts [14,18,22,23].

Previous studies found that low diversity and low *Blautia* spp. abundance in fecal samples obtained before HSCT were also associated with a higher risk of acute GvHD development [18,24,25,26]. In our study, a trend to a lower abundance of *Blautia* spp. was observed in the preH samples of the patients that subsequently developed GvHD (7.22 [0.93–23.7]) in comparison to those that did not (10.75 [2.89–16.76]). However, this difference did not reach statistical significance (*p* > 0.05), most probably due to the low number of GvHD cases (*n* = 13) included in our study, which highlights the need for much larger multicenter cohorts to identify biomarkers of GvHD susceptibility. As Fredricks [1] stated, the temporal dynamics of the gut microbiome constitute a critical determinant in delineating the causal interplay between microbial composition and the pathogenesis of GvHD. Therefore, establishing whether perturbations in the intestinal microbial ecosystem precede, coincide with, or are subsequent to GvHD onset is fundamental for inferring causality. Again, this objective necessitates further rigorously designed longitudinal investigations with high-resolution temporal sampling encompassing key clinical phases—pre-conditioning, hematopoietic engraftment, and the period of maximal GvHD vulnerability. Crucially, the identification of microbial alterations that temporally precede GvHD onset offers critical insights into differentiating putative driving or causal microbial taxa from those that appear because of the disease process.

In the past, some controversy arose on the role of *Blautia* spp. in the human gut; on one hand, this genus was associated with several potential beneficial properties for the host, but on the other hand, it was also pinpointed as a contributor for obesity. However, a recent systematic review showed that current evidence does not demonstrate the involvement of *Blautia* spp. in obesity development or progression [27]. On the contrary, several studies have shown the protective role of some *Blautia* species in the obesity and metabolic syndrome context through different mechanisms, including the amelioration of gut inflammation [28,29,30]. Additionally, it has been suggested that depletion of certain *Blautia* species in the gut ecosystem occurs in cases of infant obesity and contributes to metabolic inflammation, leading to insulin resistance [31]. The beneficial effect of *Blautia* spp. has also been observed in relation to other clinical settings, including Crohn’s disease, colorectal cancer, pouchitis, cirrhosis, hepatic encephalopathy and COVID-19, where increased fecal abundance of this genus was associated with reduced inflammation and disease severity, improving outcomes [32,33,34,35]. Consequently, the genus *Blautia* is generally considered a marker of a functional and intact intestinal barrier and good intestinal health [9].

In our study, the relative abundance of *Thomasclavelia* was significantly lower in postH1 and GvHD samples than in postH2 samples, and in postH1 than in preH samples, while that of *Anaerostipes* was also higher in preH samples than in postH1 and GvHD samples. The genus *Thomasclavelia* was created in 2023 to include some species that had been previously classified within the genera *Clostridium* or *Erysipelatoclostridium* [36]. Increases in the presence of *Thomasclavelia ramosa* (formerly known as either *Clostridium ramosum* or *Erysipelatoclostridium ramosum*), the most abundant species of this genus living as commensal in the human gut, have been associated with the beneficial and persistent impact of some probiotics in piglets [37] and humans [38]. In addition, the same species, together with *Ruminococcus gnavus* and *Blautia* spp., was found to be an influential hub species of the remission-associated microbial network in patients with ulcerative colitis, a fact that highlights their potential to control inflammation [39]. On the other hand, it should be considered that the beneficial or deleterious effect of a bacterial taxon may depend on a specific strain, concentration and host status, among other factors; in this context, *T. ramosa* has also been considered an opportunistic pathogen mainly affecting immunocompromised elderly [40]. In our study, the genus *Romboutsia* also showed a lower relative abundance in GvHD samples compared to postH2; this genus, often associated with a healthy status, is a well-known butyrate producer and has been pinpointed as a potential biomarker for the early detection of gut tumors [41]. Like *Blautia*, these genera belong to the class *Clostridia*, which has been repeatedly associated with a reduced incidence of lethal GvHD [5,22,42].

Previous works reported that fecal samples of GvHD patients were different from those of non-GvHD patients in relation to the relative abundance of, among others, the genera *Faecalibacterium, Akkermansia* and *Veillonella* [13], *Enterococcus* [18,43], *Lactobacillus* [26], *Bacteroides* and *Parabacteroides* [13,44], *E. coli/Shigella* and/or *Klebsiella* [44]. Interestingly, in our study, the genera *Bacteroides* and *Parabacteroides*, which are well-known propionate producers [45,46,47], showed a lower relative abundance in the preH samples of those patients that later developed GvHD; this is in accordance with that reported by Biagi et al. (2015) [13], who suggested that the immunomodulatory properties of propionate could exert a protective effect on GvHD development. However, since no other metataxonomic differences were found between pre-HSCT samples from patients who did and did not develop GvHD, it is not clear whether they are the cause of the increased risk of GvHD or if they only reflect the patient’s preexisting condition.

Various mechanisms have been proposed to explain how the intestinal microbiota might influence the prevention or development of GvHD [1,14,48,49]. Certain members of the microbiota could exert a protective effect by producing certain metabolites that counteract the inflammatory cascade that often occurs in patients who develop GvHD, or by competitively excluding other microorganisms that promote inflammatory processes. This appears to be the case for some members of the *Clostridia* class, which are major producers of SCFAs (especially butyric and acetic acids), among which the genus *Blautia* is particularly representative [50].

A decrease in SCFA concentration was observed after HSCT. Butyrate contributes to reinforcing the gut mucosal barrier and is a primary energy source for intestinal epithelial cells [51]; in addition, it also affects inflammatory signaling pathways, inducing regulatory T cells [52] and reducing inflammatory responses [53,54]. Butyrate also acts as a histone deacetylase inhibitor to inhibit antigen-stimulated T cells that mediate GvHD. In this regard, SCFAs produced by the gut microbiota have been shown to attenuate GvHD [55], while, conversely, low levels of intestinal butyrate and other SCFAs have been associated with the onset or increased severity of GvHD [23,56]. These data suggest a close link between butyrate-producing gut bacteria, such as *Blautia* spp., and other members of the class *Clostridia*, and a decreased risk of GvHD after HSCT. *Blautia* spp. also produces propionate, for which anti-inflammatory effects have been demonstrated [57]. Additionally, acetate, which in this work decreased after HSCT in GvHD when compared with non-GvHD patients, has been shown to reduce inflammation and improve epithelial barrier function in the gastrointestinal tract [58]. *Blautia* is also one of the most prominent acetate-producing bacteria among the autochthonous members of the gut, as demonstrated by its abundance and dominance among acetate-producing strains within the family microbiota [59,60].

Interestingly, propionate levels showed a relative increase at postH2 compared to earlier post-transplant time points. This finding may reflect the progressive recovery of the gut microbiota following HSCT. Previous studies have shown that HSCT induces a marked disruption of gut microbial composition, followed by gradual microbiota reconstitution over time [9,11,12]. This recovery may lead to the restoration of microbial metabolic activity, including SCFA production. In addition, microbiota recovery has been associated with immune reconstitution and restoration of intestinal homeostasis after HSCT [13]. In fact, propionate and butyrate have been associated with protection from chronic GvHD [61]. In our work, the concentrations of propionate, butyrate and acetate were higher among the patients that did not develop GvHD but, overall, only that of propionate reached a statistical significance when postH2 samples were compared to preH samples. The gut microbiota is known to exhibit resilience after major clinical perturbations, with progressive recovery of its metabolic functions [26]. Therefore, the increase in propionate observed at postH2 may reflect a progressive restoration of microbial activity during later stages of post-transplant recovery.

This study faces limitations as our results are from a single center and the total number of GvHD cases was relatively low. The results obtained in this project must be corroborated in subsequent multicenter studies involving a much larger number of patients; anyhow, given the correlation with the results of previous studies, at least regarding the notably lower levels of *Blautia* spp., butyrate and acetate in the feces of GvHD patients, they indicate the plausibility of new avenues that contribute to predicting the prognosis (e.g., the quantitative PCR specific for *Blautia* spp. or measuring the levels of fecal butyrate). Similarly, this information may be useful for improving the prevention and treatment of GvHD by, for example, using highly specific live biotherapeutic agents or transferring fecal material from selected donors. This approach would be based on selected components of the human intestinal microbiota with a very specific metabolism, capable of competing with those that can promote inflammatory processes and, in addition, of exerting beneficial interactions with the immune system.

In conclusion, this work showed that there are strong shifts in the fecal microbiota composition over time along with a sharp decrease in microbial diversity after the HSCT procedure; such changes were mirrored by shifts in the fecal SCFA profiles of the samples. Patients suffering intestinal GvHD show microbial signatures prior to HSCT characterized by a lower relative abundance of the genera *Blautia* and *Bacteroides* when compared to non-GvHD patients.

## 4. Materials and Methods

### 4.1. Design of the Study and Participants

This single-center, observational, and prospective study was performed in Hospital Infantil Universitario Niño Jesús (Madrid, Spain) and included patients undergoing allogeneic HSCT from January 2021 to July 2023 who fulfill the following inclusion criteria: (a) pediatric patients (up to 18 years of age); (b) patients who were scheduled for allogeneic HSCT throughout the duration of the study; (c) availability of clinical history including data on the underlying disease and treatments received; (d) patients who completed a clinical evaluation including blood analysis and, in the case of those with suspected GvHD, a rectosigmoidoscopy; and (e) explicit acceptance of parents/legal guardians of the patient to participate in the study by signing the corresponding informed consent. Exclusion criteria included the following: (a) patients with clinical instability contraindicating the previously mentioned clinical evaluation; (b) patients who refused or could not undergo the complete clinical evaluation; and (c) non-acceptance of parents/legal guardians of the patient to participate in the study. This study was approved by the Ethics Committee of the Hospital Infantil Universitario Niño Jesús (Madrid, Spain) (protocol: R-0009/21; date of approval: 14 June 2021).

Fecal samples were collected at three time points: prior to conditioning (preH), after HSCT (when engraftment occurs, postH1), and 100 days after HSCT (postH2). Furthermore, an additional sample (GvHD) was collected from patients with GvHD. In these patients, fecal samples were collected after histopathological confirmation of the diagnosis. The samples were stored at −80 °C until processing.

### 4.2. DNA Extraction

DNA was extracted from the fecal samples using the QIAamp DNA Stool Mini Kit (QIAgen, Hilden, Germany) with the modifications proposed by Lackey et al. [62]. The concentration and purity of the extracted DNA were measured using a NanoDrop One (Thermo Scientific, Madrid, Spain) spectrophotometer. The DNA samples were stored at −20 °C until processing.

### 4.3. Amplification, Sequencing of the 16S rRNA Gene and Bioinformatic Analysis

The hypervariable V3-V4 region of the bacterial 16S rRNA gene was amplified using PCR. Equimolar concentrations of the universal primers S-D-Bact-0341-b-S-17 and S-D-Bact-0785-a-A-21 were used. PCR products were pooled in equimolar DNA concentrations and ran on agarose gels. Bands were extracted and purified using the QIAEX II Gel Extraction Kit (QIAgen) and quantified with PicoGreen (BMG Labtech, Jena, Germany). Aliquots of the amplicons were sequenced using the paired-end sequencing protocol on the Illumina MiSeq sequencer (Illumina Inc., San Diego, CA, USA) at the Scientific Park of Madrid, Spain. Sequences were demultiplexed using Illumina software (version 2.6.2.3) following the manufacturer’s guidelines.

Bioinformatic analyses were conducted using a combination of QIIME 2 2021.1 and R software (version 3.5.1, https://www.r-project.org/ (accessed on 1 September 2025). The DADA2 pipeline was used for sequence cleaning and filtering: forward reads were truncated at position 285 with the first 12 nucleotides trimmed, and reverse reads were truncated at position 240 with the first nine nucleotides trimmed to discard positions with a mean nucleotide quality below Q20. Taxonomy data were assigned to amplicon sequence variants (ASVs) using the q2-feature-classifier classify-sklearn naïve Bayes classifier against the SILVA 138.1 reference database.

The Shannon and Simpson α-diversity indices were calculated using the R vegan package to estimate alpha diversity, considering both the number and evenness of microbial species, with the Wilcoxon rank-sum test used to identify statistical differences between groups. Beta diversity was studied using principal coordinate analysis (PCoA) to visualize patterns through a distance matrix. Quantitative and qualitative analyses were performed using Bray–Curtis and binary Jaccard indices, respectively. Permutational multivariate analysis of variance (PERMANOVA) with 999 permutations was employed.

### 4.4. Short-Chain Fatty Acid (SCFA) Analysis

The quantification of SCFAs in the fecal samples was performed by gas chromatography as previously described [63]. Briefly, 100 μL of a 1:10 dilution of fecal matter (wet weight/v) in phosphate-buffered saline (PBS; pH 7.4) was supplemented with 100 μL of 2-ethyl butyric acid (Sigma-Aldrich, St. Louis, MO, USA) as an internal standard (1 mg/mL in methanol) and acidified with 100 μL of 20% formic acid (*v*/*v*). The acidic mixture was then extracted with 1 mL of methanol and centrifuged for 10 min at 15,800× *g*. The supernatants were stored at −20 °C until analysis using a GC apparatus. The system comprised a 6890 GC injection module (Agilent Technologies, Santa Clara, CA, USA) with an HP-FFAP (30 m × 0.250 mm × 0.25 μm) column (Agilent Technologies), operating in split mode with a split ratio of 1:20. The injection volume was 1 μL, with injector and detector temperatures set at 240 °C and 250 °C, respectively. The column oven temperature was initially set at 110 °C, then increased at a rate of 6 °C/min to 170 °C, and subsequently at 25 °C/min to 240 °C, resulting in a total GC run time of 18 min. Helium was used as the carrier gas at a constant flow rate of 1.3 mL/min. The chromatographic system featured a flame ionization detector (FID), and data acquisition and processing were carried out using the ChemStation Agilent software Version LTS 01.11 (Agilent Technologies).

### 4.5. Statistical Analysis

Statistical analyses were conducted using R software. Differences were considered statistically significant at *p* < 0.05. Alpha diversity was assessed using both the Shannon and Simpson diversity indices, which account for the abundance and evenness of microbial species. Beta diversity was illustrated using principal coordinate analysis (PCoA) derived from distance matrices. The Bray–Curtis index was employed for quantitative analysis, while the binary Jaccard index was used for qualitative analysis. Statistical differences in beta diversity were evaluated using PERMANOVA with 999 permutations, identifying significant differences at *p* < 0.05.

## Figures and Tables

**Table 1:** Demographic characteristics and main outcomes of the study participants.

Characteristic	All Patients (*n* = 59)	Non-GvHD (*n* = 45; 76%)	GvHD (*n* = 14; 24%)	*Exitus* (*n* = 14; 24%)
Gender (Male/Female)	34/25 (58%/42%)	26/19 (58%/42%)	8/6 (57%/43%)	5/9 (36%/64%)
Median Age (years, range)	11.91 (3.50–21.22)	12.13 (3.65–21.22)	11.89 (3.50–16.36)	11.51 (6.84–17.46)

**Table 2:** Bacterial alpha diversity at each sampling point as assessed using the Shannon and Simpson indices. The values are expressed as median [IQR].

Group	Shannon Index	Simpson Index
preH	3.15 [2.58–3.73] ^#^	0.92 [0.86–0.95]
postH1	2.38 [1.70–3.37]	0.85 [0.70–0.94]
postH2	3.37 [3.00–3.71] *^†^	0.93 [0.89–0.95] ^†^
GvHD	2.00 [1.31–2.55]	0.82 [0.63–0.89]

^#^ *p* < 0.05 vs. postH1; * *p* = 0.05 vs. GvHD; ^†^ *p* < 0.001 vs. postH1 (Wilcoxon, Bonferroni corrected).

**Table 3:** Frequency of detection and relative abundance of main bacterial phyla (bold) and genera in the fecal samples from the four sampling points analyzed in this study. The *p*-values obtained from pair comparisons between the different groups are shown in Table S1.

	preH		postH1		postH2		GvHD	
	*n* (%)	median [IQR]	*n* (%)	median [IQR]	*n* (%)	median [IQR]	*n* (%)	median [IQR]
**Bacillota**	53 (100%)	86.54 [75.74–94.09]	43 (100%)	83.08 [51.89–95.45]	38 (100%)	91.2 [79.52–96.59]	5 (100%)	36.15 [31.48–89.91]
*Blautia*	53 (100%)	8.38 [2.45–17.89]	43 (100%)	0.23 [0.03–4.58]	38 (100%)	14.62 [6.36–26.13]	5 (100%)	0.02 [0.01–0.04]
*Streptococcus*	53 (100%)	2.02 [0.63–4.73]	43 (100%)	4.53 [0.43–19.65]	38 (100%)	1.5 [0.47–6.73]	5 (100%)	13.52 [3.41–25.09]
*Enterococcus *	53 (100%)	0.05 [0.02–2.04]	43 (100%)	0.6 [0.03–20.56]	38 (100%)	0.41 [0.03–1.18]	5 (100%)	0.03 [0.02–0.03]
*Ruminococcus gnavus group *	52 (98.11%)	0.4 [0.01–2.26]	41 (95.35%)	0.01 [0.01–0.25]	38 (100%)	1.32 [0.04–3.91]	5 (100%)	0.01 [<0.01–0.01]
*Agathobacter *	53 (100%)	0.01 [0.01–2.18]	42 (97.67%)	0.01 [0.01–0.04]	38 (100%)	0.18 [0.01–3.57]	5 (100%)	0.01 [0.01–0.01]
*Thomasclavelia *	46 (86.79%)	0.64 [0.07–2.09]	34 (79.07%)	0.04 [<0.01–0.49]	36 (94.74%)	1.22 [0.49–2.69]	3 (60%)	0.01 [<0.01–0.01]
*Lacticaseibacillus *	31 (58.49%)	<0.01 [<0.01–0.05]	30 (69.77%)	0.01 [<0.01–0.09]	27 (71.05%)	0.01 [<0.01–0.05]	5 (100%)	0.02 [0.02–22.58]
*Gemmiger *	51 (96.23%)	0.01 [<0.01–0.75]	38 (88.37%)	0.01 [<0.01–0.05]	37 (97.37%)	0.03 [<0.01–1.69]	5 (100%)	<0.01 [<0.01–0.01]
*Clostridium_innocuum_group *	50 (94.34%)	0.31 [0.02–1.44]	36 (83.72%)	0.06 [<0.01–0.98]	33 (86.84%)	0.41 [0.06–2.65]	4 (80%)	0.01 [<0.01–0.01]
*Faecalibacterium *	43 (81.13%)	0.05 [<0.01–2.38]	34 (79.07%)	0.01 [<0.01–0.2]	36 (94.74%)	0.51 [<0.01–3.63]	5 (100%)	0.01 [0.01–0.01]
*Anaerostipes *	50 (94.34%)	0.3 [0.01–2.13]	37 (86.05%)	0.01 [<0.01–0.06]	36 (94.74%)	0.05 [<0.01–2.03]	3 (60%)	<0.01 [<0.01–0.01]
*Romboutsia *	43 (81.13%)	0.29 [0.01–0.93]	32 (74.42%)	<0.01 [<0.01–0.05]	36 (94.74%)	0.62 [0.03–1.5]	3 (60%)	<0.01 [<0.01-<0.01]
**Pseudomonadota**	53 (100%)	0.26 [0.06–8.56]	43 (100%)	1.13 [0.13–28.94]	38 (100%)	1.21 [0.19–8.27]	5 (100%)	25.28 [10.02–63.78]
*Escherichia*/*Shigella*	50 (94.34%)	0.01 [0.01–1.91]	43 (100%)	0.01 [0.01–0.15]	38 (100%)	0.71 [0.03–3.68]	5 (100%)	0.07 [0.01–3.48]
*Klebsiella*	45 (84.91%)	0.01 [<0.01–0.02]	35 (81.4%)	0.01 [<0.01–0.02]	32 (84.21%)	0.01 [<0.01–0.03]	4 (80%)	0.01 [0.01–0.38]
*Ralstonia*	38 (71.7%)	<0.01 [<0.01–0.02]	36 (83.72%)	0.02 [0.01–0.21]	28 (73.68%)	<0.01 [<0.01–0.01]	5 (100%)	0.01 [<0.01–0.11]
**Bacteroidota**	46 (86.79%)	1.19 [<0.01–4.83]	39 (90.7%)	0.45 [0.02–4.13]	35 (92.11%)	0.41 [0.01–2.28]	4 (80%)	<0.01 [<0.01–0.03]
*Bacteroides*	42 (79.25%)	0.59 [<0.01–3.74]	31 (72.09%)	0.02 [<0.01–1.6]	31 (81.58%)	0.17 [<0.01–1.97]	3 (60%)	<0.01 [<0.01–<0.01]
*Parabacteroides*	31 (58.49%)	<0.01 [<0.01–0.39]	25 (58.14%)	<0.01 [<0.01–0.14]	23 (60.53%)	<0.01 [<0.01–0.16]	2 (40%)	<0.01 [<0.01–<0.01]
**Actinomycetota**	53 (100%)	2.16 [0.31–4.33]	43 (100%)	1.14 [0.17–5.02]	38 (100%)	1.18 [0.42–3.98]	5 (100%)	0.03 [0.03–0.07]
*Bifidobacterium*	43 (81.13%)	0.12 [<0.01–1.84]	40 (93.02%)	0.01 [<0.01–0.06]	35 (92.11%)	0.15 [<0.01–1.82]	4 (80%)	<0.01 [<0.01–0.01]
**Verrucomicrobiota**	16 (30.19%)	<0.01 [<0.01–<0.01]	20 (46.51%)	<0.01 [<0.01–<0.01]	6 (15.79%)	<0.01 [<0.01–<0.01]	0 (0%)	<0.01 [<0.01–<0.01]
Minor_phyla	48 (90.57%)	0.03 [0.01–0.12]	38 (88.37%)	0.09 [0.01–0.29]	35 (92.11%)	0.04 [0.01–0.08]	5 (100%)	0.01 [<0.01–0.09]
Minor_genera	53 (100%)	28.19 [16.05–36.21]	43 (100%)	18.5 [4.26–33.17]	38 (100%)	25.95 [14.45–33.7]	5 (100%)	19.86 [0.18–28.83]
Unclassified_genera	53 (100%)	9.57 [3.17–16.32]	43 (100%)	8.68 [1.13–19.81]	38 (100%)	11.12 [4.81–22.36]	5 (100%)	3.41 [0.51–26.91]

*n* (%): Number and percentage of samples in which the phylum/genus was detected (relative frequency of detection). The relative abundance (%) of each bacterial genus is expressed as the median and the interquartile range [IQR].

**Table 4:** Median values (IQRs) of the concentrations (μg/g) of acetate, butyrate and propionate in the fecal samples of the different study groups.

SCFAs	preH	postH1	postH2	GvHD
Acetate	2005.8 ^ad^ (836.4–3394.4)	524.7 ^ab^ (62.4–1587.6)	3048.60 ^be^ (1205.20–4866.0)	585.4 ^de^ (427.5–1257.3)
Butyrate	803.9 ^ad^ (191.00–1341.1)	21.6 ^ab^ 3.00–244.8)	796.60 ^be^ (451.20–1257.3)	3.0 ^de^ (3.0–34.7)
Propionate	477.1 ^ac^ (182.1–927.9)	64.4 ^ab^ (7.8–329.2)	821.4 ^cbe^ (391.3–1177.8)	84.0 ^e^ (7.80–262.9)

^a^ preH and postH1 values are statistically different (*p* < 0.05); ^b^ postH1 and postH2 values are statistically different (*p* < 0.05); ^c^ preH and postH2 values are statistically different (*p* < 0.05); GvHD values are statistically different from those of both ^d^ preH and ^e^ postH2 (*p* < 0.05).

## Data Availability

The 16S rRNA gene sequences analyzed in this study are available in the SRA database repository under the accession code PRJNA1405955. Further inquiries can be directed to the corresponding authors.

## Supplementary Materials

The following supporting information can be downloaded at https://www.mdpi.com/article/10.3390/ijms27052331/s1.

## Abbreviations

The following abbreviations are used in this manuscript:

HSCT	Hematopoietic stem cell transplantation
GvHD	Graft-versus-host disease
SCFA	Short-chain fatty acids
IQR	Interquartile range
