# Supplementary material for: Metataxonomic Analysis and Fatty Acid Profiling of Feces from Children Undergoing Hematopoietic Stem Cell Transplantation
Source: Int J Mol Sci. 2026 Mar 2;27(5):2331. doi: 10.3390/ijms27052331 (PMC12984869; doi:10.3390/ijms27052331)
Supplement: Supplementary file 1 [file ijms-27-02331-s001.zip › Captions of Figure and Tables-revised.pdf]

Figure S1: Heat-map showing pairwise relationships between SCFA's concentrations, sampling group, GvHD and *exitus*. Each cell reports the two-sided Mann–Whitney p value (first line) and the median [Q1, Q3] (second and third lines) for the first (A) and second (B) groups compared in each column. Background colors denote significance: beige $0.01 \leq p < 0.05$, orange $0.001 \leqslant p < 0.01$, red $p < 0.001$; white indicates $p \geqslant 0.05$).

Table S1: p-values obtained when the relative abundance of the main bacterial phyla (bold) and genera (italics) detected in each study group were compared with the other groups.

Table S2: Concentrations (median [IQR]) of the short-chain fatty acid, expressed as μg/g, stratified by sampling groups and clinical outcomes.
