# Supplementary material for: Metataxonomic Analysis and Fatty Acid Profiling of Feces from Children Undergoing Hematopoietic Stem Cell Transplantation
Source: Int J Mol Sci. 2026 Mar 2;27(5):2331. doi: 10.3390/ijms27052331 (PMC12984869; doi:10.3390/ijms27052331)
Supplement: Supplementary file 1 [file ijms-27-02331-s001.zip › Table S1-revised.pdf]

| **Table S1.** *P*-values obtained when the relative abundance of the main bacterial phyla (bold) and genera (italics) detected in each study group were compared with the other groups. Bonferroni was the P value adjustment method used for every phylum/genus comparison. | | | | | | |
|---|---|---|---|---|---|---|
| | ***p*-values** | | | | | |
| | preH *vs* postH1 | preH *vs* postH2 | preH *vs* GvHD | postH1 *vs* postH2 | postH1 *vs* GvHD | postH2 *vs* GvHD |
| **Bacillota** | 1.000 | 1.000 | 1.000 | 0.870 | 1.000 | 1.000 |
| *Blautia* | 0.001 | 0.273 | 0.020 | <0.001 | 0.404 | <0.001 |
| *Streptococcus* | 0.970 | 1.000 | 0.520 | 1.000 | 1.000 | 0.600 |
| *Enterococcus* | 0.180 | 1.000 | 1.000 | 1.000 | 0.470 | 0.550 |
| *Ruminococcus gnavus group* | 0.275 | 0.609 | 0.649 | 0.002 | 1.000 | 0.174 |
| *Agathobacter* | 0.180 | 1.000 | 0.300 | 0.250 | 1.000 | 0.830 |
| *Thomasclavelia* | 0.031 | 0.750 | 0.137 | <0.001 | 1.000 | 0.022 |
| *Lacticaseibacillus* | 1.000 | 1.000 | 0.150 | 1.000 | 0.700 | 0.450 |
| *Gemmiger* | 1.000 | 1.000 | 1.000 | 0.870 | 1.000 | 0.890 |
| *Clostridium innocuum group* | 0.910 | 1.000 | 0.350 | 0.410 | 1.000 | 0.220 |
| *Faecalibacterium* | 0.823 | 1.000 | 1.000 | 0.086 | 1.000 | 0.932 |
| *Anaerostipes* | 0.001 | 1.000 | 0.034 | 0.221 | 0.349 | 0.193 |
| *Romboutsia* | 0.007 | 0.607 | 0.080 | <0.001 | 1.000 | 0.007 |
| **Pseudomonadota** | 0.410 | 1.000 | 0.070 | 1.000 | 0.850 | 0.240 |
| *Escherichia/Shigella* | 1.000 | 0.177 | 1.000 | 0.047 | 1.000 | 1.000 |
| *Klebsiella* | 1.000 | 1.000 | 1.000 | 1.000 | 1.000 | 1.000 |
| *Ralstonia* | 0.006 | 1.000 | 0.831 | <0.001 | 1.000 | 0.272 |
| **Bacteroidota** | 1.000 | 1.000 | 1.000 | 1.000 | 1.000 | 1.000 |
| *Bacteroides* | 1.000 | 1.000 | 1.000 | 1.000 | 1.000 | 1.000 |
| *Parabacteroides* | 1.000 | 1.000 | 1.000 | 1.000 | 1.000 | 1.000 |
| **Actinomycetota** | 1.000 | 1.000 | 0.012 | 1.000 | 0.007 | 0.001 |

| | | | | | | |
|---|---|---|---|---|---|---|
| *Bifidobacterium* | 1.000 | 1.000 | 1.000 | 1.000 | 1.000 | 0.840 |
| **Verrucomicrobiota** | 1.000 | 0.397 | 0.988 | 0.017 | 0.372 | 1.000 |
| *Minor_phyla* | 1.000 | 1.000 | 1.000 | 1.000 | 0.940 | 1.000 |
| *Minor_genera* | 0.270 | 1.000 | 1.000 | 0.670 | 1.000 | 1.000 |
| *Unclassified_genera* | 1.000 | 1.000 | 1.000 | 0.820 | 1.000 | 1.000 |
