# Supplementary material for: Metataxonomic Analysis and Fatty Acid Profiling of Feces from Children Undergoing Hematopoietic Stem Cell Transplantation
Source: Int J Mol Sci. 2026 Mar 2;27(5):2331. doi: 10.3390/ijms27052331 (PMC12984869; doi:10.3390/ijms27052331)
Supplement: Supplementary file 1 [file ijms-27-02331-s001.zip › Table S2-revised.pdf]

**Table S2.** Concentrations (median [IQR]) of the short-chain fatty acids, expressed as μg/g, stratified by sampling groups and clinical outcomes.								
	n	**Acetic**	**Butyric**	**Caproic**	**Isobutyric**	**Isovaleric**	**Propionic**	**Valeric**
All patients	153	1587.6 [521.3 - 3048.6]	330.2 [52.4 - 931.6]	20.4 [20.4 - 20.4]	9.95 [9.95 - 66.0]	72 [11.9 - 180.6]	357.6 [61.5 - 862.8]	8.85 [8.85 - 28.8]
**Sampling group**								
preH	55	2005.8 [836.4–3394.4]	803.9 [191.4–1341.1]	20.4 [20.4–20.4]	9.9 [9.9–93.6]	78.7 [11.9–184.8]	477.1 [182.1–927.6]	8.8 [8.8–41.4]
postH1	53	524.7 [62.4–1587.6]	21.6 [3.0–244.8]	20.4 [20.4–20.4]	9.9 [9.9–9.9]	11.9 [11.9–83.4]	64.4 [7.8–329.2]	8.8 [8.8–8.8]
postH2	41	3048.6 [1205.2–4866.0]	796.6 [451.2–1257.3]	20.4 [20.4–20.4]	9.9 [9.9–112.8]	138.6 [67.2–256.8]	821.4 [391.3–1177.8]	8.8 [8.8–54.6]
GvHD	4	585.4 [427.5–617.1]	3.0 [3.0–34.7]	20.4 [20.4–20.4]	9.9 [9.9–9.9]	11.9 [11.9–11.9]	84.0 [7.8–262.9]	8.8 [8.8–8.8]
**GvHD**								
No	124	1762.2 [607.4 - 3309.6]	430.9 [82.7 - 960.0]	20.4 [20.4 - 20.4]]	9.9 [9.9 - 87.6]	81 [11.9 - 191.4]	455.4 [109.0 - 917.4]	8.8 [8.8 - 45.6]
Yes	31	585.4 [99.6 - 2194.4]	108.5 [3.0 - 629.1]	20.4 [20.4 - 20.4]	9.9 [9.9 - 9.9]	11.9 [11.9 - 94.8]	179.8 [7.8 - 568.4]	8.8 [8.8 - 8.8]
Exitus								
No	121	1762.2 [585.0 - 3388.8]	451.2 [103.8 - 1070.5]	20.4 [20.4 - 20.4]	9.9 [9.9 - 87.6]	76.3 [11.9 - 202.5]	477.1 [109.0 - 931.2]	8.8 [8.8 - 36.7]
Yes	32	811.7 [88.2 - 1979.1]	73.5 [3.0 - 457.8]	20.4 [20.4 - 20.4]	9.9 [9.9 - 9.9]	11.9 [11.9 - 112.5]	166.0 [7.8 - 412.2]	8.8 [8.8 - 8.8]
**Sampling group_GvHD**								
PreH_No	42	2005.8 [1242.7 - 3535.9]	865.6 [239.2 - 1343.3]	20.4 [20.4 - 20.4]	9.9 [9.9 - 103.7]	117.0 [11.9 - 204.9]	591.9 [257.6 - 990.6]	8.8 [8.8 - 54.9]
PreH_Yes	13	1392.9 [240.2 - 2964.6]	361.8 [43.6 - 903.3]	20.4 [20.4 - 20.4]	9.9 [9.9 - 33.6]	11.9 [11.9 - 125.4]	194.4 [38.1 - 482.4]	8.8 [8.8 - 8.8]

PostH1_No	41	602.0 [215.7 - 1735.2]	52.4 [3.0 - 253.2]	20.4 [20.4 - 20.4]	9.9 [9.9 - 9.9]	11.9 [11.9 - 93.0]	125.6 [7.8 - 576.0]	8.8 [8.8 - 8.8]
PostH1_Yes	12	131.2 [20.9 - 549.8]	3.0 [3.0 - 104.0]	20.4 [20.4 - 20.4]	9.9 [9.9 - 9.9]	11.9 [11.9 - 14.9]	33.1 [7.8 - 192.8]	8.8 [8.8 - 8.8]
PostH2_No	34	3179.1 [1014.2 - 4718.1]	774.0 [452.2 - 1249.8]	20.4 [20.4 - 20.4]	31.0 [9.9 - 112.5]	141.0 [27.7 - 252.5]	842.1 [394.9 - 1087.1]	8.8 [8.8 - 55.1]
PostH2_Yes	7	2346.0 [1830.1 - 5322.6]	981.0 [437.5 - 1178.8]	20.4 [20.4 - 20.4]	9.9 [9.9 - 88.4]	90.6 [73.5 - 303.4]	768.6 [536.3 - 1423.8]	8.8 [8.8 - 8.8]
GvHD_Yes	4	585.4 [427.5 - 617.1]	3.0 [3.0 - 34.7]	20.4 [20.4 - 20.4]	9.9 [9.9 - 9.9]	11.9 [11.9 - 11.9]	84.0 [7.8 - 262.9]	8.8 [8.8 - 8.8]
