# Supplementary figures and images for: Metataxonomic Analysis and Fatty Acid Profiling of Feces from Children Undergoing Hematopoietic Stem Cell Transplantation
Source: Int J Mol Sci. 2026 Mar 2;27(5):2331. doi: 10.3390/ijms27052331 (PMC12984869; doi:10.3390/ijms27052331)

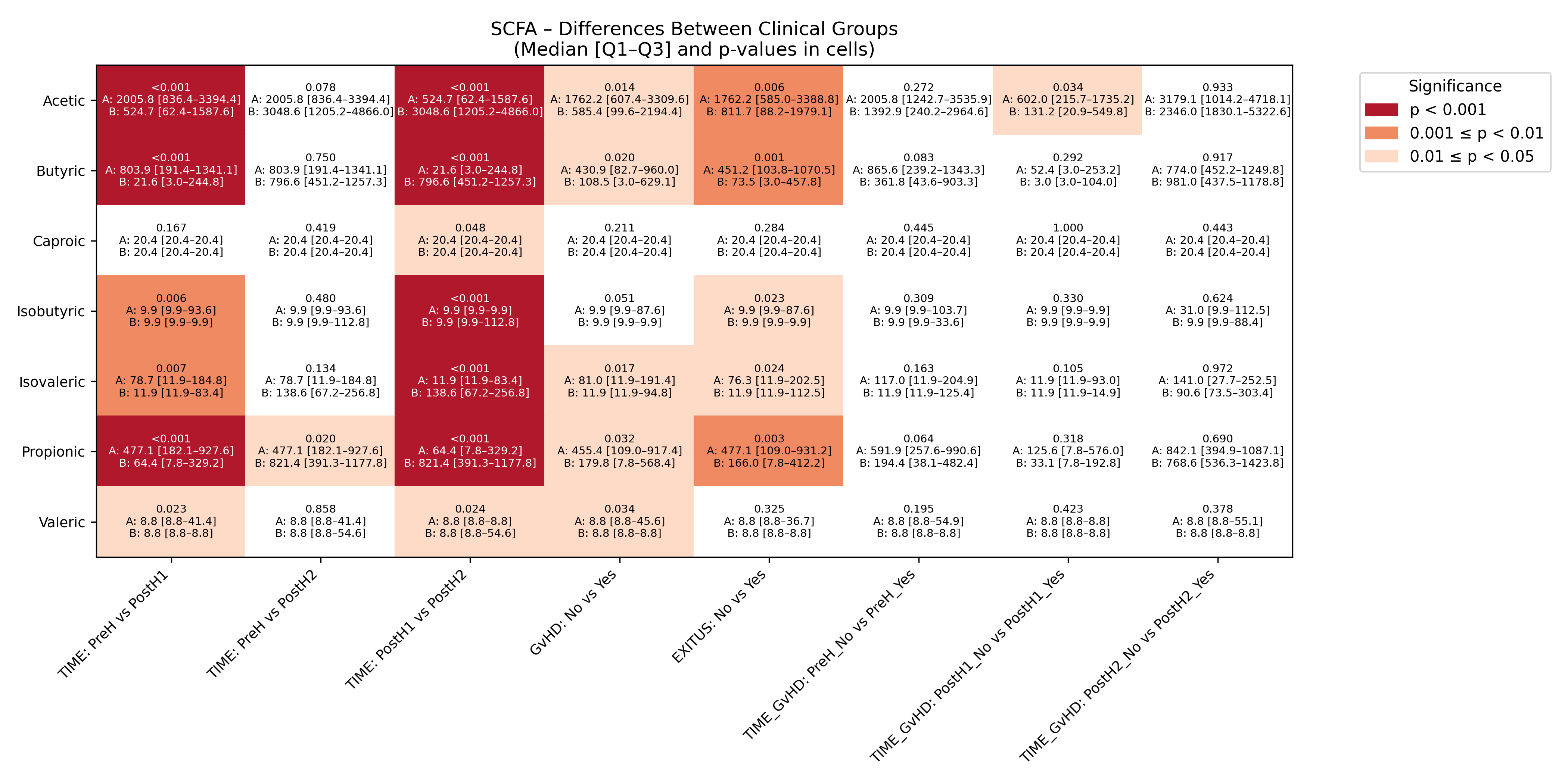

Supplement: Supplementary file 1 [file ijms-27-02331-s001.zip › Figure S1.tiff]
